# Co‐occurrence patterns in diagnostic data

**DOI:** 10.1111/coin.12317

**Published:** 2020-04-12

**Authors:** Marie Ely Piceno, Laura Rodríguez‐Navas, José Luis Balcázar

**Affiliations:** ^1^ Computer Science Department Universitat Politècnica de Catalunya (UPC) Barcelona Spain; ^2^ Life Sciences Department Barcelona Supercomputing Center (BSC) Barcelona Spain

**Keywords:** clan decomposition, exploratory data analysis, Gaifman graphs

## Abstract

We demonstrate how graph decomposition techniques can be employed for the visualization of hierarchical co‐occurrence patterns between medical data items. Our research is based on Gaifman graphs (a mathematical concept introduced in Logic), on specific variants of this concept, and on existing graph decomposition notions, specifically, graph modules and the clan decomposition of so‐called 2‐structures. The construction of the Gaifman graphs from a dataset is based on co‐occurrence, or lack of it, of items in the dataset. We may select a discretization on the edge labels to aim at one among several Gaifman graph variants. Then, the decomposition of the graph may provide us with visual information about the data co‐occurrences, after which one can proceed to more traditional statistical analysis.

## 1 INTRODUCTION

We propose to employ decomposition techniques on Gaifman graphs as an exploratory data analysis approach on medical data. The Gaifman graphs record the co‐occurrences of data items in datasets and, then, graph decompositions may provide valuable information that is not directly observable on the data, since they display a hierarchical visualization of the co‐occurrences.

Graphical descriptions add enormously to the interpretability of the outcomes of data analysis in many fields, including medical data, where we can cite the work on the Diasesome graph, for one.^1^, ^2^ On the other hand, highly frequent co‐occurrences of data items have been a target for several types of data mining frameworks for decades, in all types of data, including medical data.^3^ Most commonly studied notions for this sort of analysis are frequent sets and variants thereof, such as frequent closed sets or association rules.^4^, ^5^ In most cases, frequent set mining actually returns a large textual list; then, a visualization of this result can allow us to achieve a better understanding of the data, as well as to discover implicit and potentially useful information. Examples of tools that aim to explain visually frequent patterns or association rules are PyramidViz^6^ or arulesViz.^7^, ^8^

Despite the large body of literature, these notions are not really reaching out to end users, mainly due to the difficulty of finding explanatory descriptions. In fact, one of the main setbacks is that, mathematically speaking, the results of these notions of data analysis are found in spaces of huge dimensionality, and their reductions to 2D or 3D plots almost never offer enough interpretability.

Gaifman graphs are a notion originated in mathematical studies of the logical structures that, actually, support the relational database model. We have argued in previous work^9^ that these graphs can be advantageously employed for exploratory data analysis via their decomposition in terms of so‐called *clans*,^10^ either in their original form or in one of its variants. We are preparing a separate publication^11^ containing a theoretical study of precise mathematical properties of these graph decompositions, relating them to closure spaces and explaining the main algorithmics behind our analyses.

These decompositions of Gaifman graphs have the potential to reveal “co‐occurrence” patterns or, alternatively, “incompatibility” patterns, since they relate together data items that, pairwise, appear together somewhere in the whole dataset. Generalizations of the notion allow us to adjust the co‐occurrence thresholds so as to account for particularly frequent joint occurrences, or for different intervals of co‐occurrence counts, as we shall see. This is achieved through specific variants of Gaifman graphs, namely, exponential and linear Gaifman graphs and their decompositions; also, we extend here our considerations to a novel, shortest‐path‐based variant of Gaifman graphs.^12^

We focus here on showing how these combinatorial techniques are applicable to medical data corresponding to joint diagnostics of patients. We believe that our visualizations can act in a useful way complementing existing statistical approaches, for example, by pointing out specific pairs of elements, possibly conditioned to other elements, whose correlation studies could be candidates for priority analysis.

This submission is archival version of previous conference publications, which included section 2,^9^ sections 3, 4.1, and part of 4.2 (presented at the CBMS conference to which this issue is devoted), and the rest of section 4.^12^

## 2 GAIFMAN GRAPH DECOMPOSITION

Initially, we will work with standard Gaifman graphs and their modular decomposition. The theory of 2‐structures and their clans will be provided later on, after explaining the need to use them for our generalizations of Gaifman graphs. In graph theory, the modular decomposition of a graph is a process that decomposes the vertices of the graph into sets called modules.

Definition 1 Given a graph, a set of vertices *X* is a module if, for each vertex *y*∉*X*, either every member of *X* is connected with *y* or every member of *X* is not connected with *y*.

The modules of a graph can be seen as subgraphs of the original graph. Of course, we have some trivial modules such as the singleton items and the complete set of vertices. In order to obtain a tree‐like form in the decomposition, we work with a special kind of modules, the so‐called strong modules, which avoid overlapping:

Definition 2 Two sets *X* and *Y* overlap if the sets *X*∩*Y*, *X*∖*Y*, and *Y*∖*X* are all three nonempty. Equivalently, *X* and *Y* do *not* overlap if and only if they are either disjoint or a subset of one another.

Definition 3 A module *M* is a strong module of a graph if it does not overlap with any other module.

The modular decomposition of a graph is simply the set of strong modules, depicted in a tree‐like manner so that every strong module is associated with a single vertex within its parent, that is, the smallest strong module containing it. We describe first some simple examples below and then move on to those corresponding to our diagnostic data patterns.

### 2.1 Gaifman graphs

Gaifman graphs are logical mathematical structures whose basic notion is pretty simple. Given a first‐order relational structure where the values appearing in the tuples of the relations ${ℛ}_{i}$ come from a fixed universe 𝒰, its corresponding Gaifman graph has the elements of 𝒰 as vertices, and the edges (*x*,*y*) for *x*≠*y* are determined exactly when *x* and *y* appear together in some tuple $t\in {ℛ}_{i}$ for some ${ℛ}_{i}$.

Thus, it could be applied directly on relational dataset where the relations ${ℛ}_{i}$ will be the tables in the database, the tuples $t\in {ℛ}_{i}$ will be those rows in the tables, the set of vertices 𝒰 will be determined by all possible attribute values, and the edge (*x*,*y*) will be determined exactly when the attribute values *x* and *y* appear together in some row.

It is important to point out that often, in practice, each column of a table has its own semantics and, even if a data value seems to superficially coincide in occurrences at different columns, it might mean different things (eg, the same string yes as a value of columns named homeowner and haschildren might prompt us to consider them as different values). In addition, there are other times where it is better to take these values as one and the same, that is, regardless of the attribute that they represent. We assume that some previous preprocessing has taken care of, ensuring that semantically different attribute values are literally different as well.

Example 1 Let us consider a very small relational dataset on the universe 𝒰 = {*a*_0_,*a*_1_,*b*_0_,*b*_1_,*b*_2_,*b*_3_} conformed by the tuples:

$$\begin{array}{ll} t_{0}:\;a_{0}\;b_{0} & \\ t_{1}:\;a_{0}\;b_{1} & \\ t_{2}:\;a_{0}\;b_{2} & \\ t_{3}:\;a_{0}\;b_{3} & \\ t_{4}:\;a_{1}\;b_{0} & \\ t_{5}:\;a_{1}\;b_{1} & \\ t_{6}:\;a_{1}\;b_{2} & \end{array}$$

**FIGURE 1:**
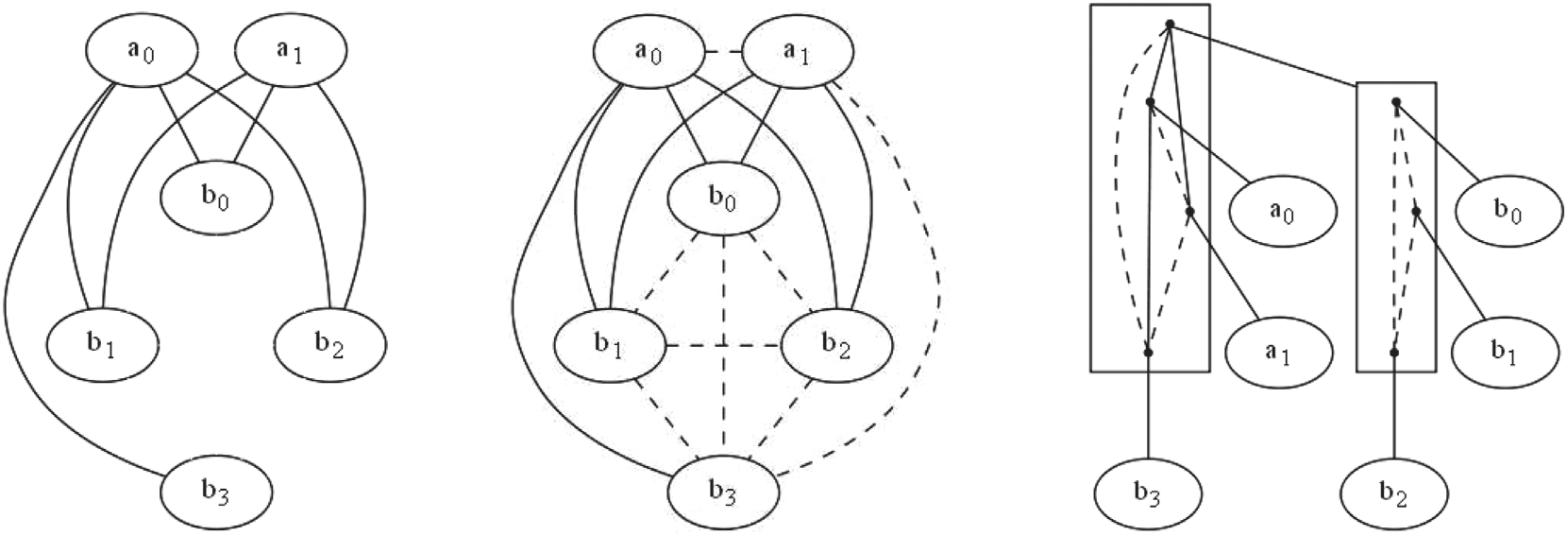
A standard Gaifman graph of Example 1, its natural completion and a standard graph decomposition

The Gaifman graph that represents its co‐occurrences is shown in Figure 1 (left). According to the definition, the vertices are all the possible attribute values and the edges link pair of attributes values that appear together in some row. An alternative drawing, that will fit the 2‐structure‐based approach described shortly, is the so‐called “natural completion” of the Gaifman graph, shown in Figure 1 (center), where attribute values that sometimes appear together are joined by solid lines, while attribute values that never appear together are joined by broken lines, thus leading to a complete graph with two classes of edges.

The modular decomposition of the Gaifman graph in Figure 1 (left) is shown in Figure 1 (right). We keep the edge colors as are in its natural completion, shown in Figure 1 (center). Boxes correspond to sets that are strong modules, and dots within them to subsets that are also strong modules. All along the whole decomposition, trivial (single‐item) modules are indicated by a link to the vertex they consist of, represented with an elliptic node; nontrivial ones are linked instead to a new box describing the internal structure of the module, in terms of the strong modules it has again as proper subsets.

At the rightmost box, three of the *b* values are connected by broken lines, which means that they never co‐occur together: indeed, as different values of the same attribute, no row can have two of them. In the other box, the top node is a condensed version of the module formed by *b*_0_, *b*_1_, and *b*_2_, and it is connected with solid lines to both *a*_0_ and *a*_1_, meaning that all the ways of pairing one of the *a*'s with one of these *b*'s do appear in the data. Like the attribute values *b*_*i*_, the values *a*_*i*_ do not appear together in any row and they appear connected by a broken line. More interestingly, we could have expected to see the attribute value *b*_3_ in the same module of the rest of the values *b*_*i*_; however, the fact that it appears in the higher module points out to us that, unlike the other *b*_*i*_ values, there is not any co‐occurrence of *b*_3_ with *a*_1_. Of course, there are no co‐occurrences of *b*_3_ with the other *b*_*i*_'s either. That is, the items *b*_0_, *b*_1_, and *b*_2_ “behave equally”: all are connected to *a*_0_ and *a*_1_ and all are disconnected to *b*_3_; this is why they conform a module. However, *b*_3_ cannot join them since it “behaves differently” with respect to *a*_0_ and *a*_1_, as it co‐occurs with *a*_0_ in the data but not with *a*_1_.

We also may extend, in a natural way, the construction of the Gaifman graph from a transactional dataset. Transactional datasets, also known as “market‐basket datasets”,^5^ consist of a sequence of transactions, each of which consists, in turn, of a set of items. Then, the Gaifman graph from a transactional dataset will have as set of vertices 𝒰, all the possible items found in the transactions, and the edge (*x*,*y*) will be determined exactly when the items *x* and *y* appear together in some transaction.

If we want to see a transactional dataset as a relational dataset, each item will be an attribute with binary values: the presence or absence of it in the original tuple. In most practical cases, those zeros representing absence of an item abound, yet we are only interested in items being jointly present, so the zeros are not informative. This is the reason of using the Gaifman graph transactional construction as we have just described.

Example 2 Let us consider a very small dataset, quite similar to that shown in our earlier work,^7^ on the universe 𝒰={a,b,c,d,e} conformed by the transactions:

$$\;\begin{array}{lll} t_{1} & :\;a\;b\;c & \\ t_{2} & :\;a\;e & \\ t_{3} & :\;a\;c\;d & \\ t_{4} & :\;d\;e & \end{array}$$

**FIGURE 2:**
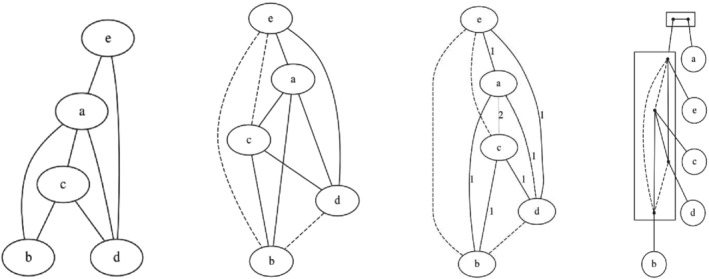
A standard Gaifman graph of Example 2, its natural completion, a labeled variant and a standard graph decomposition

The Gaifman graph that represents its information is shown in Figure 2 (left). According to the definition, each vertex of the graph represents an item, and edges link pairs that appear together in one of the transactions. Its natural completion Gaifman graph variant is shown in Figure 2 (center‐left), where items that sometimes appear together are joined by solid lines, while items that never appear together are joined by broken lines, again, leading to a complete graph with two classes of edges.

The modular decomposition of the graph in Figure 2 (left) is displayed in the rightmost diagram of Figure 2. The topmost box corresponds to the set of all the vertices and, inside it, both dots correspond to a strong module each.

Gaifman graphs record co‐occurrence (or lack of it) among every pair of attribute values or items of the universe 𝒰, but there are cases where it is useful to have quantitative information about the co‐occurrences.

In the previous example, we may see the edges labeled by their multiplicity, that is, the number of transactions that contain both endpoint items, in Figure 2 (center‐right); it is intuitive to represent different labels with different colors. In this way, some pairs do not appear together and the corresponding edges are labeled with zero and represented by dashed edges, those pairs that appear together exactly once are labeled 1 and represented by black lines, and the pair that appears two times is labeled 2 and represented by a gray line. As a result, we have a graph with three classes of edges, where the modular decomposition is not enough anymore. Therefore, we work with 2‐structures and their decompositions, which naturally generalize modular decompositions to more than two equivalence classes of edges.

### 2.2 Generalizations of Gaifman graph

We will employ some variants of Gaifman graphs, proposed in our earlier work^7^ and based on simple quantitative discretizations. Our first variant is as follows. For a threshold *k* (a nonzero natural number) a thresholded Gaifman graph is a graph in which each labeled edge is classified according to its number of co‐occurrences, as follows: if the number in the label is above the threshold *k*, the edge goes into one equivalence class (represented in our diagrams by a solid line), whereas if the number of co‐occurrences of the edge is less than or equal to the threshold, then the edge belongs to the other equivalence class (and a broken line is used to represent it). In this case, we still have two equivalence classes of edges. The standard Gaifman graph corresponds to the case *k*=0.

Further variants rely on label discretization. Back to the labeled Gaifman graph variation as in Figure 2 (center‐right), taken at face value, we find as many equivalence classes as different multiplicities are there, but also we may define the equivalence classes by some sort of discretization process. We work here with two different discretizations, namely, linear and exponential, and with yet another variant based on shortest paths.

Equivalence classes are denoted indexed, as ${ℰ}_{i}$, where index *i* plays a role in the definition of ${ℰ}_{i}$ itself. Let *c*_*x*,*y*_ be the number of co‐occurrences of items *x* and *y*. In the linear Gaifman graphs, the equivalence classes of edges (*x*,*y*) are determined according to an interval size: $(x,y)\in {ℰ}_{i}$ if and only if $i=\left\lceil c_{x,y}/n\right\rceil$, being *n*>0 the assigned interval size.

In the exponential Gaifman graphs, the width of the equivalence classes grows in an exponential way; thus, $(x,y)\in {ℰ}_{i}$ if and only if $i=\left\lceil \log_{2}c_{x,y}+1\right\rceil$ 1.

Finally, in the shortest‐paths Gaifman graph, each edge is labeled according to the length of the shortest path that connects its endpoints in the standard Gaifman graph. All these variants, linear, exponential, and shortest paths, can be combined with the threshold version in the obvious manner. In fact, all these generalizations of Gaifman graphs are actually 2‐structures, as we are going to explain next. They can be decomposed as such, in terms of their strong clans, that generalize strong modules to more than two equivalence classes. However, we find handy to keep referring to them as graphs.

### 2.3 2‐structures and their decomposition

The notion of modular decomposition is not enough to handle adequately the generalizations of Gaifman graphs that we proposed; therefore, we work with a more general notion, 2‐structures and their clans.^10^

Definition 4 A 2‐structure is complete graph on some universe with an equivalence relation among its edges.

For a 2‐structure, we say that a subset C⊆𝒰 is a clan, informally, if all the members of *C* are indistinguishable among them by nonmembers. That is, whenever some *x*∉*C* “can distinguish” between *y*∈*C* and *z*∈*C*, in the sense that the edge (*x*,*y*) is not equivalent to the edge (*x*,*z*), then *C* is not a clan. Formally:^10^

Definition 5 Given 𝒰 and an equivalence relation y∈ℰ⊆((U×U)×(U×U)) on the edges of the complete graph on *U*. Then C⊆𝒰 is a clan when:

∀x∉C∀y∈C∀z∈C((x,y),(x,z))∈ℰ.

Thus, two members of a clan cannot be connected by edges in different equivalence classes to the same vertex outside the clan. The notion naturally generalizes that of modules, which are clans in terms of two equivalence classes (existing edges and absent edges). Similarly, we have trivial clans: the singleton items and the universe by itself. And, also, by focusing on strong clans, we get a tree‐like form in the decomposition.^10^

Definition 6 A clan *C* is a strong clan of a graph if it does not overlap with any other clan.

## 3 GAIFMAN GRAPH OF MEDICAL DATA

To analyze a dataset using our graphical tool, we construct the 2‐structure that represents its information using one of the variants of Gaifman graph, then we apply the clan decomposition method. Thus, the corresponding 2‐structure will have as vertices all the attribute values or items (depending on whether we work on a relational or a transactional dataset) and a setup of edges according to the sort of Gaifman graph chosen. Often, these graphs have huge amounts of vertices; in order to get a humanly understandable, smaller but representative version of the graph, we choose to work with those vertices that appear into the transactions more frequently than a determined threshold.

The dataset is made up of hospitalizations where the transactions correspond to patient encounters; thus, each transaction of the dataset is a set of diagnostics, medical treatments, and, possibly, other, related information.

This dataset was provided to us by the Hospital de la Santa Creu i Sant Pau, under a collaboration agreement between that institution and UPC. This is a public hospital located in Barcelona, with about 430K visits and 40K admissions per year. The dataset contains information of all hospitalizations for the years 2015 and 2016, in the format sent for billing purposes to the public funding agency. It consists of a sequence of (Excel‐like) rows: they correspond to patients and, in each, there are, organized in columns, diagnostics and treatments corresponding to that patient, encoded in ICD‐9‐CM^2^.

Additional information (such as provenance, gender, etc) is also present in the data but not taken into account in our analyses. The data are fully anonymized and include a total of 79 534 rows. The data make a distinction between primary information (diagnostics and treatments) and a varying number of secondary ones but, again, our analyses do not take this difference into account yet.

Most observations have only a handful of items: lots of patient encounters just include a couple of diagnostics; however, the total number of potential diagnostics is 5637, growing to 7741 if treatments are also considered and to 8250 if patient conditions are considered too. Thus, considering the set as relational would result in a huge dimensionality, with vast amounts of zeros. Hence, we chose to see our dataset as transactional, each row consisting of a set of diagnostics, procedures, and/or conditions, and we construct the Gaifman graph as indicated in the previous section for the transactional case.

The study is divided into three parts. In each part we analyze the result of applying the clan decomposition method on different Gaifman graph variations. In the first part we work with the exponential variant of the Gaifman graphs; we focus first on diagnostics only, and then on diagnostics and treatments together. In the second part, we work with the linear variant of the Gaifman graphs, adding the patient condition to the analysis. And, in the last part, we expand on the first and second sections with the shortest‐path Gaifman graph variant.

## 4 INTERPRETING MEDICAL GAIFMAN GRAPH VARIANTS

As indicated above, we cannot display graphically all the different diagnostic attribute values of the medical dataset; thus, for each visualization process, we follow an attitude akin to that of frequent set mining.^5^, ^13^ That is, we give a minimum frequency threshold, so that items that appear less often than the threshold are not taken into account for the visualization.

### 4.1 Exponential Gaifman graph

**FIGURE 3:**
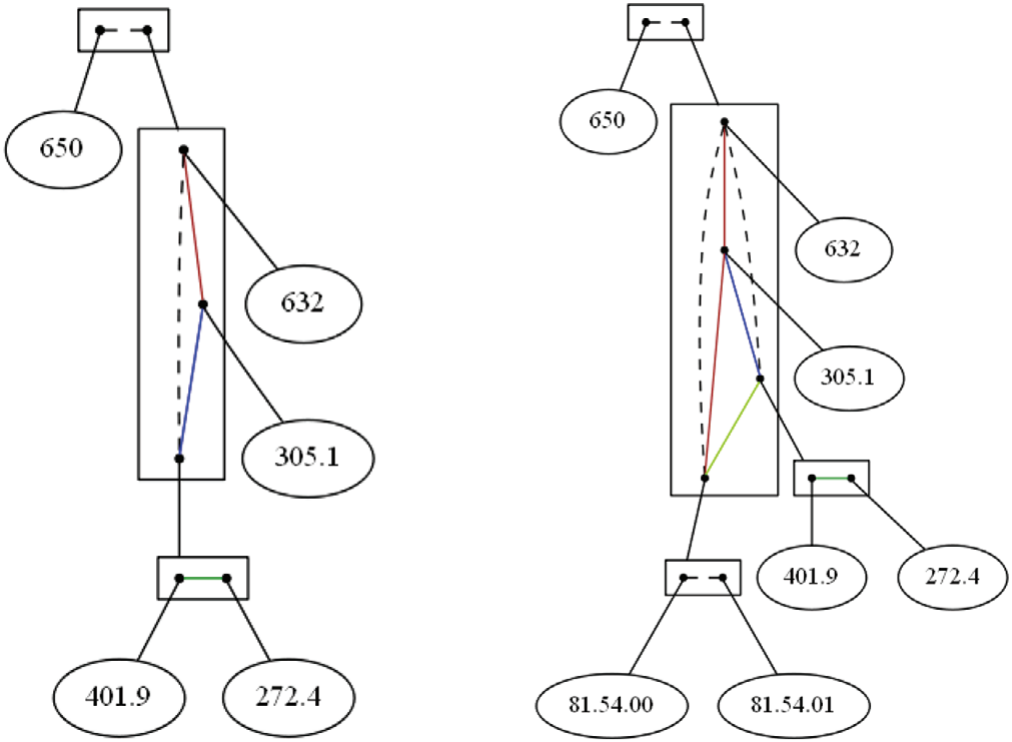
Diagnostics appearing at least 100 times and diagnostics and treatments appearing at least 100 times

Figure 3 (left) shows us the decomposition of the exponential Gaifman graph of those diagnostics above a frequency threshold of 100. Those values are:

650: *Normal delivery*
632: *Missed abortion*
305.1: *Tobacco use disorder*
401.9: *Unspecified essential hypertension*
272.4: *Other and unspecified hyperlipidemia*

We impose a co‐occurrence threshold set at eight transactions, that is, one 10 000th of the dataset size (thus, all the items co‐occurring eight times or less are consider as though they do not co‐occur often enough).

At the top of the figure we see that 650 *Normal delivery* does not appear often enough together with any of the other items represented in the figure because 650 is connected with a broken line to the box corresponding to the remaining diagnostics. Inside the larger box we find, as the bottom vertex, a clan conformed by 272.4 *Other and unspecified hyperlipidemia* and 401.9 *Unspecified essential hypertension*, since they have the same “behavior” with the remaining diagnostics, that is, the diagnostics 272.4 and 401.9 co‐occur each one around the same number of times with the remaining diagnostics. We name this clan as hypertension clan. We find that these two items are highly connected, with around 11 000 co‐occurrences.

The edges from the item 305.1 *Tobacco use disorder* are in different equivalence classes because tobacco disorder co‐occurs with the members of the hypertension clan around 2000 times, while with the item 632 *Missed abortion* just a dozen of times. We also have that the edge connecting 632 *Missed abortion* with the hypertension clan is a broken line since they co‐occur less than eight times.

In the next example, we consider diagnostic and treatments, having 7741 different values. Thus, we decided to work with those diagnostics and treatments appearing more than 100 times, and also with 8 as co‐occurrence threshold. Comparing with the previous example, we find two new values corresponding to procedural items:

81.54.00: *Total knee replacement (left)*
81.54.01: *Total knee replacement (right)*

Figure 3 (right) shows us the decomposition. Additional to the previous decomposition, we find a new clan conform by the treatments related to the replacement of knees. They are connecting by a broken line that indicates that they are incompatible, since it is hardly ever the case that both knees are replaced at once.

We also see that there are very few joint occurrences from 632 *Missed abortion* with the bottom node corresponding to the knee replacement procedure clan. Among them, the knee replacement procedure clan and the items in the hypertension clan (401.0 *Unspecified essential hypertension* and 272.4 *Other and unspecified hyperlipidemia*) appear jointly around 100 times, and, finally, diagnostic 305.1 *Tobacco use disorder* with the knee replacement procedure clan co‐occurs not too significant number of times, about a dozen times each (same as 305.1 and 632). That is why we can find the different equivalence classes on the edges.

### 4.2 Linear Gaifman graph

Until here, we have illustrated the process and some of the possible results applying the data analysis approach based on the decomposition of exponential Gaifman graphs on the medical dataset. In this section we consider another variation, the use of linear Gaifman graph.

In the first example, we take into account those diagnostics appearing more 80 times. We apply the clan decomposition method on the linear Gaifman graphs with an interval size of 1000, which means that the co‐occurrences of the data will be divided into intervals of that size.

Thus, the items involved in the first decomposition are:

650: *Normal delivery*
250.00: *Diabetes mellitus without mention of complication, type II or unspecified type, not stated as uncontrolled*
401.9: *Unspecified essential hypertension*
305.1: *Tobacco use disorder*
278.00: *Obesity unspecified*
272.4: *Other and unspecified hyperlipidemia*
634.92: *Spontaneous abortion complete without complication*
632: *Missed abortion*

**FIGURE 4:**
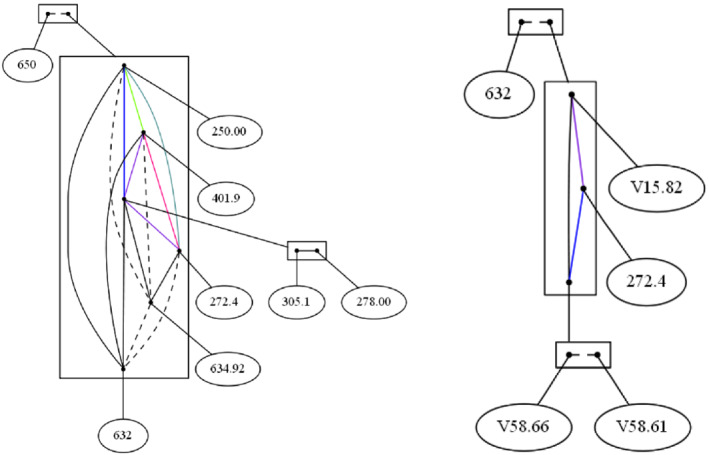
Linear: diagnostics appearing at least 80 times and diagnostics, treatments, and patient conditions appearing at least 250 times

In Figure 4 (left) we have at the top of the figure the item 650 disconnected with all of the others, that is, 650 *Normal delivery* has no any co‐occurrence with any other diagnostic.

We find a clan conformed by the items 305.1 *Tobacco use disorder* and 278.00 *Obesity unspecified*. Both of them co‐occur around 500 times. In fact, those items that co‐occur less than 1000 times are connected with edges in the same equivalence class than the edge from 305.1 to 278.00. The item 250 *Diabetes mellitus* co‐occurs with the tobacco‐obesity clan more than 1000 times but less than 2000 times, while 401.9 *Unspecified essential hypertension* and 272.4 *Other and unspecified hyperlipidemia* co‐occur with the same clan around 2000 times, and the clan co‐occurs just few times with the items 634.92 *Spontaneous abortion complete without complication* and 632 *Missed abortion*.

In the next example we also include patient conditions. As the data are coding using ICD‐9‐CM, the item name of the patient conditions values start as “V…”. In this case, we only take into account those items appearing more than 250 times. In this way, the items involve in the decomposition are:

632: *Missed abortion*
272.4: *Other and unspecified hyperlipidemia*
V15.82: *Personal history of tobacco use*
V58.66: *Long‐term (current) use of aspirin*
V58.66: *Long‐term (current) use of anticoagulants*

We set a threshold of co‐occurrences (namely, 500) below which items are considered as not occurring together frequently enough. Figure 4 (right) shows the result of apply the decomposition on the linear Gaifman graph version of this graph with an interval size of 1000. We obtain a clan conformed by V58.66 *Long‐term (current) use of aspirin* and V58.61 *Long‐term (current) use of anticoagulants*, they are connected by a broken line since they co‐occur less than 500 times, around 300 times, not often enough according to our threshold.

We also see that this clan, the anticoagulant clan nodes, co‐occurs more frequently with 272.4 *Other and unspecified hyperlipidemia* than with V15.82 *Personal history of tobacco use*. And the items that co‐occur more frequently than any others are the nodes 272.4 *Other and unspecified hyperlipidemia* and V15.82 *Personal history of tobacco use*. That is, the number of people who have hyperlipidemia and take anticoagulants is greater than people who have a history of smoking and take anticoagulants, while it is more frequent to have cases with history of tobacco use and having hyperlipidemia. At the top of the decomposition we find the clan 632 *Missed abortion*, since it does not co‐occur together often enough with any of these diagnostics.

### 4.3 Shortest path Gaifman graph

We propose to explore a shortest path version of the Gaifman graph variations. The decomposition on the shortest path version of the Gaifman graph provides us with complementary information about the data behavior.

**FIGURE 5:**
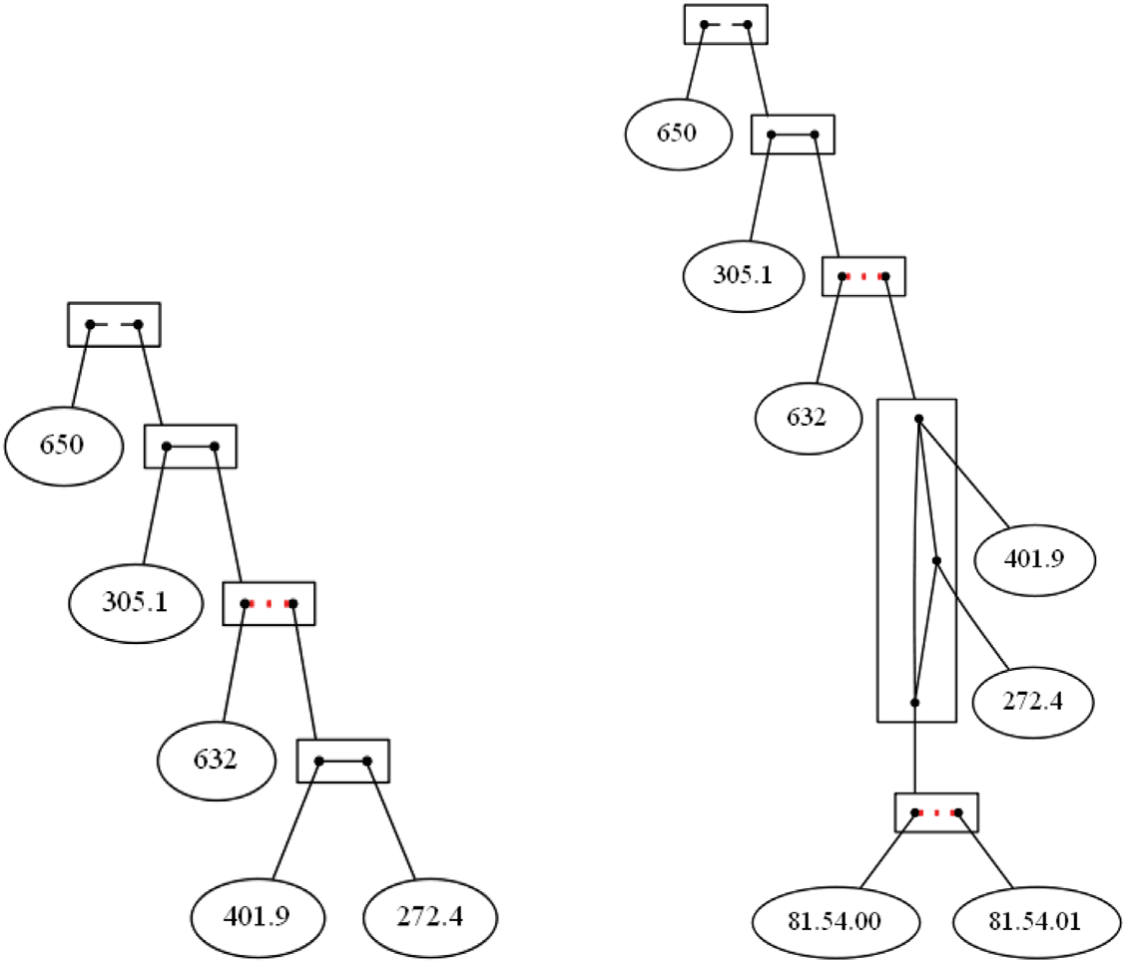
Shortest path: diagnostics appearing at least 100 times and diagnostics and treatments appearing at least 100 times

In the first example we work with those diagnostics that appear more than 100 times with a co‐occurrence threshold of 8. In Figure 5 (left) we verify that the item 650 *Normal delivery* is not connected with any other item, as in the examples of exponential and linear variants. On the contrary, the item 305.1 *Tobacco use disorder* is connected with all of the remaining items. We can also verify that the hypertension clan, formed by the items 272.4 *Other and unspecified hyperlipidemia* and 401.9 *Unspecified essential hypertension* is not directly connected to 632 *Missed abortion*, we may confirm this in Figure 3 (left).

In Figure 5 (right), we have the shortest path decomposition too but adding the treatments that appear more than 100 times and also with a co‐occurrence threshold of 8. We verify again that the item 650 *Normal delivery* is disconnected to the remaining items and the item 305.1 *Tobacco use disorder* is directly related with all the remaining items.

In this cases, additional to the previous decomposition, we find a new clan conformed by knee replacement items, 81.54.00 *Total knee replacement (left)* and 81.54.01 *Total knee replacement (right)*. The knee replacement items may co‐occur directed with the hypertension items but, inside of the clan, they are connected by a dotted line since the knee replacement procedures are not directly related.

Finally, we verify that the item 632 *Missed abortion* is not directly related with the hypertension items and knee replacement clan, we may confirm this in Figure 3 (right).

We also analyze the shortest path of those diagnostics that appear more than 80 times. Thus, the items involved in this new decomposition are:

650: *Normal delivery*
305.1: *Tobacco use disorder*
278.00: *Obesity unspecified*
272.4: *Other and unspecified hyperlipidemia*
632: *Missed abortion*
634.92: *Spontaneous abortion complete without complication*
401.9: *Unspecified essential hypertension*
250.00: *Diabetes mellitus without mention of complication, type II or unspecified type, not stated as uncontrolled*

**FIGURE 6:**
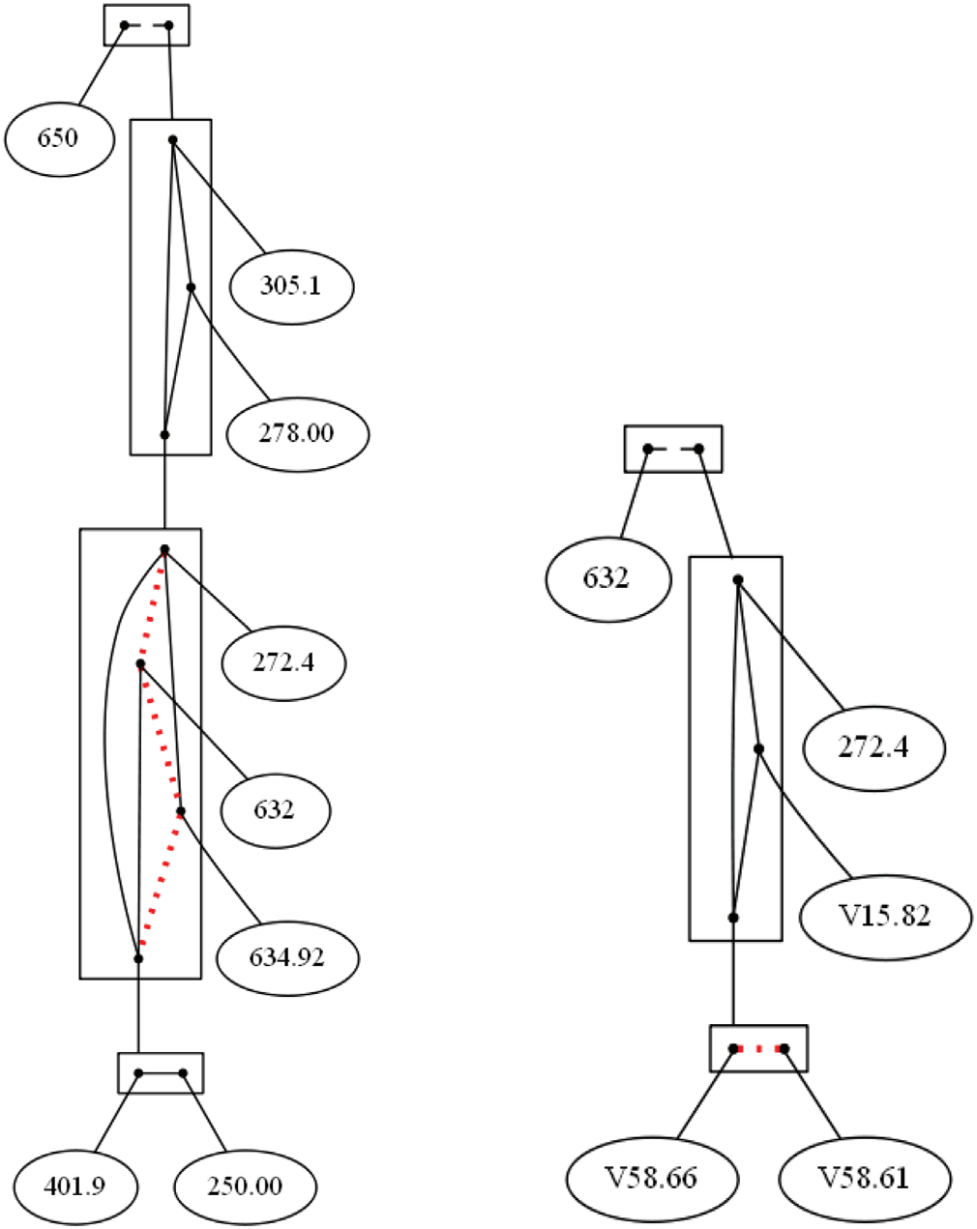
Shortest path: diagnostics appearing at least 80 times and diagnostics, treatments, and patient conditions appearing at least 250

In Figure 6 (left) we find again the item 650 *Normal delivery* disconnected to the remaining items. The items 305.1 *Tobacco use disorder* and 278.00 *Obesity unspecified* form a clan that co‐occur at least one time with any of the other items. In the large box we find a clan where the length path to go from one item to another is one or two.

In the bottom of the figure we find a clan conformed by the items 401.9 *Unspecified essential hypertension* and 250.00 *Diabetes mellitus*, they are in the same clan because they have the same behavior, both of them not co‐occur with the same items, and also we may see that they appear together since they are directly connect. We may think that the items 272.4 *Other and unspecified hyperlipidemia* and 401.9 *Unspecified essential hypertension* could be in a clan but it is not because there is a case where the item 401.9 *Unspecified essential hypertension* and 632 *Missed abortion* co‐occur but missed abortion does not have any co‐occurrence with 272.4 *Other and unspecified hyperlipidemia*.

Comparing this decomposition to the linear Gaifman graph version in Figure 4, we found one circumstantial difference. The items 632 *Missed abortion* and 272.4 *Other and unspecified hyperlipidemia* are connected since there is one transaction that contains them together, whereas in the shortest path Gaifman graph these two items are not directly related; thus, to go from 632 *Missed abortion* to the item 272.4 *Other and unspecified hyperlipidemia* implies a two length path. That is because in this example we are not working with the co‐occurrence threshold.

Back on the example in Figure 4 (right), we apply the decomposition of its shortest path Gaifman graph, as result we get the decomposition shown in Figure 6 (right). That is, in this analysis we work with diagnostics, treatments, and patient conditions that appear at least 250 times with a co‐occurrence threshold of 500. As you can see the resulting figure is quite similar, except for the type of the edges, that is, except for the equivalence relation to which they belong. At the top of the figure we reaffirm that the item 632 *Missed abortion* is not connected with any other item, will the items into the large box are directly connected, they used co‐occur together. Finally, the items V58.66 *Long‐term (current) use of aspirin* and V58.61 *Long‐term (current)* are connected by a dotted line because the shortest path to go from one to another has two as length, you can see in Figure 4 (right) that they are disconnected.

### 4.4 Run time overview

As the present work is focused on applying our proposal on the described medical dataset, the discussion of the details of the algorithms behind our system, and of their complexity, is out of the limited scope of this article. All the issues corresponding to the rich theory behind our proposal, the algorithms developed, and their complexity analysis will be discussed in depth in a forthcoming article.^11^

However, for the sake of completeness, we give here the running times spent in each of the decompositions presented here, to show that they are short enough for practical usage and that our current algorithms are sufficiently efficient (instead, our early experiments with naïve algorithmics turned out to be far too slow as soon as the number of vertices reached an interesting value). We report all these running times in Table 1.

**TABLE 1:** Running time of the decompositions

Gaifman graph	Data	Threshold	Nodes	Time (ms)
Exponential	Diagnostics	100	5	87.77
Exponential	Diagnostics and treatments	100	7	218.16
Linear (1000)	Diagnostics	80	8	363.83
Linear (1000)	Diagnostics, treatments, and patient conditions	250	5	39.3135
Shortest path	Diagnostics	100	5	129.74
Shortest path	Diagnostics and treatments	100	7	394.08
Shortest path	Diagnostics	80	8	663.59
Shortest path	Diagnostics, treatments, and patient conditions	250	5	35.8184

## 5 CONCLUSIONS

In this work we demonstrate a novel application of Gaifman graphs and their decomposition, providing a general visualization of the data behavior that could be used as a tool to complement statistical approaches.

Through this work we have illustrated the process and some of the possible results of applying the data analysis approach based on the tree decomposition of Gaifman graphs on this medical dataset. However, as in many other exploratory data analysis frameworks, we may not have luck in the decomposition of a given dataset, that is, we can find cases where the decomposition of the Gaifman graph does not have other than trivial clans, or that the decomposition has few so large substructures, providing us little or no information about the data.

Indeed, in order to obtain the previous results, we had to carefully observe the general behavior of the data. In general, at the moment, the human brain is essential during the exploration of interesting parameter settings.

That said, we believe that our visualizations can act in a useful way complementing the statistical approaches, as an example among many others, it could point to the user specific pairs of elements possibly conditioned to other elements, whose correlation studies could be candidates for priority analysis. By itself, on the other hand, our approach did not provide any interesting results when we directly applied standard concepts of quantitative pattern mining as support or confidence thresholds.^4^, ^5^

For the visualization part, we have resorted to the existing tool, the commonly used GraphViz,^14^ which was chosen due to its easy configuration, but we can imagine systems of graphic description much more powerful. In future contributions, we would like to offer self‐descriptive, more informative, perhaps even animated, visualizations that trained medical staff can immediately capture. A series of additional avenues for future research open up quite immediately. For example, it might make sense to explore, using our type of decompositions, the aforementioned Diasesome graph.^1^, ^2^ In fact, the decomposition method is not limited to Gaifman graphs.

In a completely different line, the construction of datasets like the one we work on is not so simple. Initially, many diagnoses are expressed as natural language expressions, and ICD coding of information is a separate and subsequent process, often performed by specialized people or even outsourced to companies^3^. A graphical tool trained in frequent concurrent diagnoses can help accelerate this type of process by offering common options for automatic completion and/or by checking for double verification of the rare ones, which could be either correct or the result of coding errors (like prostate surgery along with normal delivery as an extreme example).

## Funding Information

Partially supported by European Research Council (ERC) under the European Union's Horizon 2020 research and innovation programme, grant agreement ERC‐2014‐CoG 648276 (AUTAR); by grant TIN2017‐89244‐R from Ministerio de Economia, Industria y Competitividad, and by Conacyt (México). We acknowledge unfunded recognition 2017SGR‐856 (MACDA) from AGAUR (Generalitat de Catalunya).

## Footnotes

^1^
In earlier work,^9^ we have employed instead $i=\left\lfloor \log_{2}c_{x,y}\right\rfloor$; but this formula leads to mixing in the same classes the multiplicities zero and one, and now we prefer to avoid that.

^2^

https://en.wikipedia.org/wiki/International_Statistical_Classification_of_Diseases_and_Related_Health_Problems

^3^

https://en.wikipedia.org/wiki/International_Statistical_Classification_of_Diseases_and_Related_Health_Problems
